# High increase in levels of lipoprotein(a) in plasma of patients with rheumatoid arthritis after COVID‐19

**DOI:** 10.1002/art.42374

**Published:** 2022-12-08

**Authors:** David Stahl, Ruth L. Esser, Carolin Brück, Jan Thiele, Veronica Di Cristanziano, Carola tho Pesch, David M. Kofler

**Affiliations:** ^1^ Division of Rheumatology and Clinical Immunology Department of Internal Medicine Faculty of Medicine University Hospital Cologne University of Cologne and Center for Molecular Medicine Cologne University of Cologne and Center for Integrated Oncology Aachen Bonn Cologne Duesseldorf; ^2^ Division of Rheumatology and Clinical Immunology Department of Internal Medicine Faculty of Medicine University Hospital Cologne University of Cologne; ^3^ Institute of Virology Faculty of Medicine University Hospital Cologne University of Cologne; ^4^ Division of Rheumatology and Clinical Immunology Department of Internal Medicine Faculty of Medicine University Hospital Cologne University of Cologne and Center for Molecular Medicine Cologne University of Cologne Cologne, Germany

*To the Editor*:

Levels of lipoprotein(a) [Lp(a)] are elevated in plasma of patients with rheumatoid arthritis (RA) and are associated with an increased risk of developing atherosclerosis and coronary artery disease (1). Recently, a possible link between Lp(a) levels and venous thromboembolism (VTE) in patients with COVID‐19 has been discussed. The risk of VTE and cardiovascular disease is increased after SARS–CoV‐2 infection (2). Some but not all studies have shown that elevated Lp(a) levels during the acute phase of COVID‐19 are strongly correlated with an increased risk of VTE (2, 3). To date, little is known about Lp(a) levels in RA patients with previous SARS–CoV‐2 infection.

In this retrospective analysis, we compared Lp(a) plasma levels in patients with RA who had a prior diagnosis of COVID‐19 within the past 2 years (n = 22) with those who did not have a prior COVID‐19 diagnosis (n = 142). Ethics approval was not required as this retrospective analysis used clinical and laboratory data obtained from a clinical routine database. Age, sex, and RA serostatus did not differ significantly between patients with previous SARS–CoV‐2 infection and patients without previous SARS–CoV‐2 infection. Interestingly, RA patients diagnosed as having COVID‐19 within 365 days before the study start (n = 14) had significantly higher Lp(a) plasma levels compared to patients without a previous COVID‐19 diagnosis (median 90.5 nmoles/liter [interquartile range (IQR) 43.25–149.50 nmoles/liter] versus 15.0 nmoles/liter [IQR 7.0–71.0 nmoles/liter], respectively; *P* = 0.015 for pairwise comparison by Dunn‐Bonferroni test) (Figure 1A). In contrast, Lp(a) levels in RA patients with a COVID‐19 diagnosis more than 365 days before the study start (median 15.5 nmoles/liter [IQR 6.75–35.5 nmoles/liter]) were not significantly different from Lp(a) levels in patients without a prior COVID‐19 diagnosis (Figure 1A). Of note, we observed a negative correlation between Lp(a) plasma levels and time since COVID‐19 diagnosis (r = –0.564; Spearman's ρ = 0.006) (Figure 1B). In our study, serum Lp(a) levels did not differ between patient groups with different immunosuppressive treatments. Furthermore, Disease Activity Score using the C‐reactive protein level (DAS‐CRP), serum CRP levels, and the serum concentrations of other lipids (cholesterol, high‐density lipoprotein, low‐density lipoprotein, triacylglyceride) were not associated with recent COVID‐19 diagnosis.

**Figure 1:**
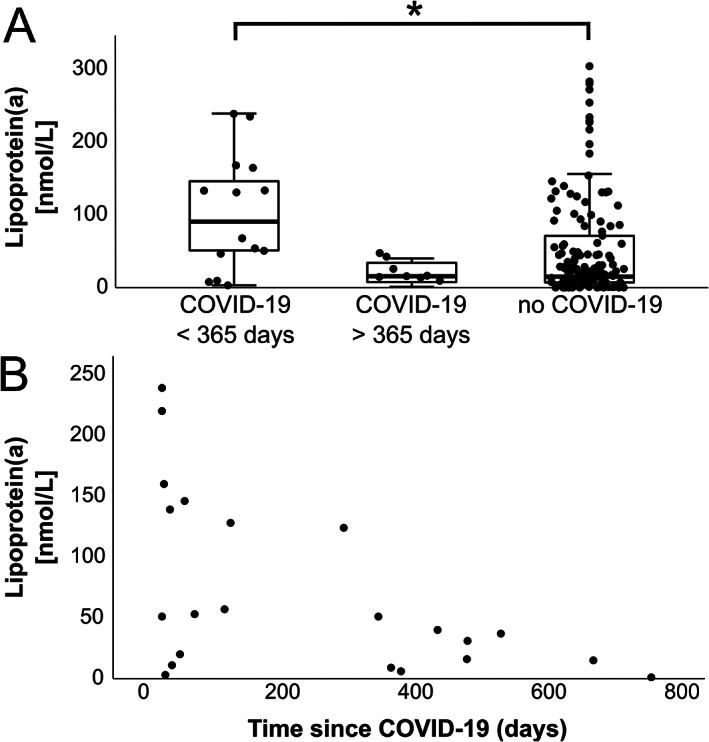
**A**, Plasma levels of lipoprotein(a) [Lp(a)] in patients with rheumatoid arthritis (RA) diagnosed as having COVID‐19 within the preceding 365 days of the analysis (<365 days since study start; n = 14), those diagnosed as having COVID‐19 more than 365 days before study start (>365 days; n = 8), or those who had no history of COVID‐19 (n = 142) (*P* = 0.014 by Kruskal‐Wallis test). * = *P* = 0.015 by Dunn‐Bonferroni pairwise test. Results are shown as box plots, in which circles represent individual samples, boxes represent the upper and lower interquartile range (IQR), lines inside the boxes represent the median, and whiskers represent 1.5 times the upper and lower IQR. **B**, Negative correlation between plasma Lp(a) levels and time since COVID‐19 diagnosis among RA patients with a prior COVID‐19 diagnosis (r = –0.564; Spearman's ρ = 0.006).

To our knowledge, this study is the first to report high and long‐lasting (up to 1 year) elevations of Lp(a) levels in plasma of RA patients after COVID‐19. However, our results do not allow any conclusions on the causality of elevated Lp(a) levels in RA patients. It remains unknown if Lp(a) levels were increased by COVID‐19, or if they were already elevated before SARS–CoV‐2 infection and led to a higher risk of COVID‐19. Elevated Lp(a) has been discussed as a potential risk factor for COVID‐19, but no clear link between Lp(a) levels and COVID‐19 risk has been established yet (3). Importantly, Lp(a) levels in plasma have been previously shown to decrease during the acute phase of Epstein‐Barr virus (EBV) infection and to normalize 4 months after EBV infection (4). A temporary increase in Lp(a) levels detected after SARS–CoV‐2 infection has not been observed after other infections.

## Supporting information

Disclosure Form
